# Supplementary material for: HOXB5 Overexpression Is Associated with Neuroendocrine Differentiation and Poor Prognosis in Prostate Cancer
Source: Biomedicines. 2021 Jul 26;9(8):893. doi: 10.3390/biomedicines9080893 (PMC8389587; doi:10.3390/biomedicines9080893)
Supplement: Supplementary file 1 [file biomedicines-09-00893-s001.zip › Supplementary compressed file proof.pdf]

Supplementary information

Supplementary Table 1 Clinicopathologic characteristics of 128 PCa patients who were treated with radical prostatectomy.

Number of cases	128
Age (years), mean ± SD, (range)	66.0 ± 5.8 (50-77)
Follow-up periods (months), mean ± SD, (range)	40.6 ± 25.3 (1-107)
PSA concentration at diagnosis (ng/ml), mean ± SD, (range)	10.5 ± 8.7 (3.2-82.3)
Pathological T stage	
pT2	105 (82.1%)
pT3	23 (17.9%)
Gleason score	
6	5 (3.9%)
7	79 (61.7%)
8	18 (14.1%)
9	26 (20.3%)
Gleason grade	
1	5 (3.9%)
2	40 (31.2%)
3	46 (36.0%)
4	18 (14.1%)
5	26 (20.3%)
D'Amico classification	
low	14 (10.9%)
Intermediate	66 (51.5%)
High	48 (37.5%)
PSA recurrence	
No	85 (66.4%)
Yes	43 (33.5%)

PCa: prostate cancer, PSA: prostate specific antigen

Supplementary Table 2 Clinicopathologic characteristics of 74 with metastatic PCa with androgen deprivation therapy

Number of cases	74
Age (years), mean ± SD, (range)	73 ± 7.0, (52-86)
Follow-up periods (months), mean ± SD, (range)	35 ± 36.4, (1-166)
PSA concentration at diagnosis (ng/ml), mean ± SD, (range)	768.8 (4.7-18740)
Gleason score	
7	15 (20%)
8	20 (27%)
9	32 (44%)
10	7 (10%)
Metastasis sites	
Lymph node	21 (28%)
Bone	60 (81%)
Lung	10 (14%)
PSA recurrence	
No	30 (40.5%)
Yes	44 (59.4%)
Time to CRPC (months), mean ± SD, (range)	36.1 ± 31.7, (2-130)

PCa: prostate cancer, PSA: prostate specific antigen

Supplementary figure 1

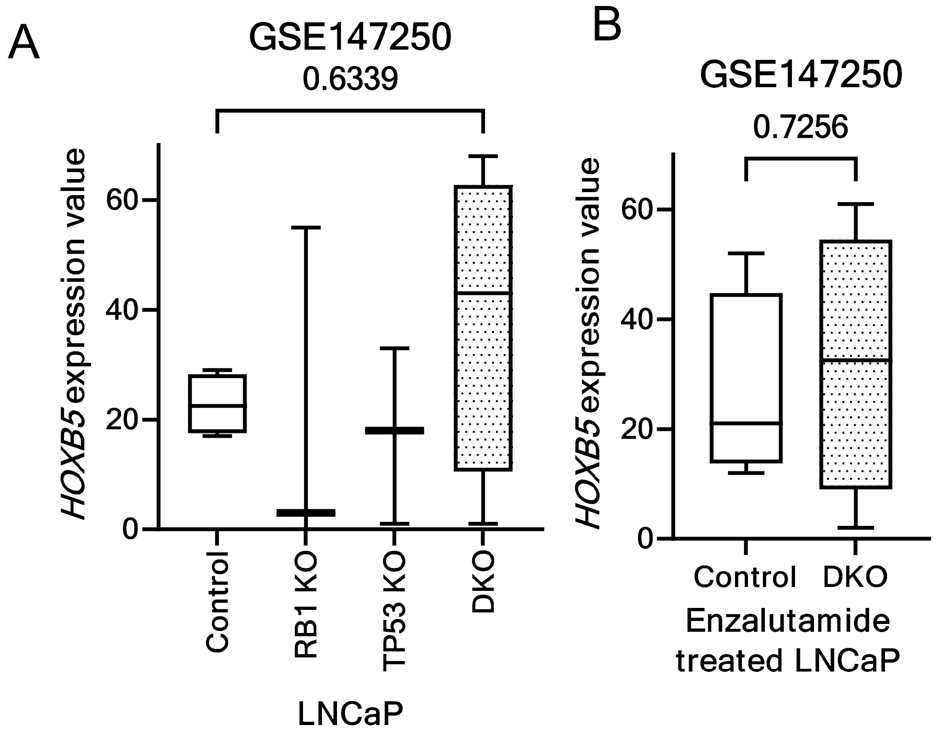

Supplementary figure 1 The expression of HOXB5 from GSE147250 (A) HOXB5 expression in control, *RB1* knockout, *TP53* knockout, and double (*RB1/TP53*) knockout cells in LNCaP. KO: knockout, DKO: double knockout (B) HOXB5 expression in control, and double (*RB1/TP53*) knockout cells in LNCaP treated with enzalutamide. DKO: double knockout
